# Identification of Small Nucleolar RNA SNORD60 as a Potential Biomarker and Its Clinical Significance in Lung Adenocarcinoma

**DOI:** 10.1155/2022/5501171

**Published:** 2022-06-07

**Authors:** Hongwei Zhou, Yibing Yao, Yan Li, Nannan Guo, Huanhuan Zhang, Zhikuan Wang, Yingtai Chen, Guanghai Dai

**Affiliations:** ^1^Chinese PLA Medical School, Beijing 100853, China; ^2^Department of General Medicine, The Fourth Medical Center of PLA General Hospital, Beijing 100048, China; ^3^Department of Oncology, Air Force Medical Center, PLA, Beijing 100142, China; ^4^Hebei North University, Zhangjiakou 075000, Hebei Province, China; ^5^Department of Thoracic Surgery, The Fourth Medical Center of PLA General Hospital, Beijing 100048, China; ^6^Senior Department of Oncology, The Fifth Medical Center of PLA General Hospital, Beijing 100071, China; ^7^Department of Thoracic Surgery, Beijing Aerospace General Hospital, Beijing 100076, China

## Abstract

Non-small-cell lung cancer (NSCLC) is the leading cause of cancer deaths in the world and often diagnosed at an advanced stage, so it is urgent to explore the pathogenesis and new diagnostic biomarkers. Accumulated evidences suggested that small nucleolar RNAs (snoRNAs) played a key role in the development and progression of NSCLC. To examine differential expression snoRNA profile and identify snoRNAs with clinical significance in lung adenocarcinoma (LUAD), The Cancer Genome Atlas (TCGA) LUAD RNA sequencing dataset was used to investigate differential expression snoRNA signatures and compared with snoRNA PCR array analysis in pair-matched LUAD tissues. The diagnostic ability of SONRD60 was assessed using a receiver operating characteristic (ROC) curve. The Kaplan-Meier method was used to plot survival curves. Univariate and multivariate Cox regression analyses were used to investigate the prognostic effect of SNORD60 expression on LUAD. The results showed that SNORD60 was a significantly upregulated snoRNA after intersection analysis in LUAD cases. SNORD60 has 74.2% sensitivity and 75.3% specificity for the diagnosis of LUAD. Increased SNORD60 expression was linked with lymph node metastases and the TNM stage (*P* < 0.05). Pathological T category and lymph node metastases were independent prognostic factors for overall survival in a multivariate Cox regression study. Our findings demonstrated that SNORD60, a small nucleolar RNA, has an oncogenic function in LUAD and might be used as a new early diagnostic biomarker for LUAD.

## 1. Introduction

Lung cancer is the leading cause of cancer-related deaths in China and worldwide, and the second most commonly diagnosed malignancy throughout the world, with approximately 2.2 million new cases in 2020 [1, 2]. Non-small-cell lung cancer (NSCLC) accounts for about 85% of lung cancer cases, with the most common subtype being lung adenocarcinoma (LUAD) [3]. Despite the great efforts to improve LUAD treatment, the survival rate remains unsatisfactory [4, 5].

Small nucleolar RNA (snoRNA) is a class of small noncoding RNAs widely distributed in the nucleoli of eukaryotic cells [6]. In recent years, with the progress of high-throughput sequencing, evidence is accumulating that snoRNA is dysregulated and involved in the development and progression of various cancers [7, 8]. Some studies have reported that snoRNAs can serve as prognostic biomarkers for cancer. However, their prognostic value in LUAD remains unknown [9–11].

This study examined snoRNA signatures based on the LUAD RNA sequencing dataset of The Cancer Genome Atlas (TCGA) and snoRNA PCR array. SNORD60 expression level was verified by quantitative real-time PCR in pair-matched LUAD tissues, and the relationship between SNORD60 expression and clinical parameters was examined. In addition, we analyzed the prognostic value of SNORD60 for overall survival. This study may provide potential diagnostic markers for LUAD.

## 2. Materials and Methods

### 2.1. Acquisition of RNA Sequencing Dataset

The transcriptome sequencing dataset (HTSeq-Counts) and clinical data of LUAD were retrieved from TCGA website (https://portal.gdc.cancer.gov) [12], and the file filter settings are shown in Supplementary Figure S1. Samples were excluded from the study based on the following criteria: (1) duplicate samples from the same patient; (2) the patient lacked survival parameters or an RNA sequencing dataset; (3) the patient had insufficient clinical data; and (4) the patient had a history of neoadjuvant therapy. Three hundred and twelve LUAD patients and 31 control cases were included in the following study. The demographic and clinicopathological characteristics of TCGA LUAD cohort are shown in Table S1 in the Supplementary Material.

### 2.2. Clinical Specimens and Patients

Surgical specimens were acquired from 15 LUAD patients who underwent lobectomy at the Fourth Medical Center of PLA General Hospital and Beijing Aerospace General Hospital between May 1, 2021, and February 17, 2022. Tumor tissues were resected intraoperatively from the surrounding lung parenchyma, and matched noncancerous lung tissues were collected from the same patients at a location away from their tumors. The World Health Organization's Lung Cancer Categorization was used to derive histopathologic classification. The TNM classification and the International Staging System for Lung Cancer [13, 14] were used to define surgical-pathological staging. The clinicopathologic characteristics of 15 LUAD patients are summarized in Table S2 in the Supplementary Material. None of the patients had adjuvant chemotherapy or radiation prior to surgery. Surgically resected tissues were frozen at −80°C. The Ethics Committees of the Fourth Medical Center of PLA General Hospital and Beijing Aerospace General Hospital approved the protocols utilized in our investigation.

### 2.3. snoRNA Profiling in Surgically Resected LUAD Tissues

Three pair-matched surgically resected tissues were used to obtain total RNA. OD260/280 measurements on the NanoDrop® ND-1000 (NanoDrop, US) were used to evaluate the purity and concentration of RNA. Denaturing agarose gel electrophoresis was used to test RNA integrity. First-strand cDNA was synthesized using the rtStar™ First-Strand Synthesis Kit (Cat# AS-FS-001, Arraystar, US), cDNA was mixed with Arraystar SYBR® Green qPCR Master Mix (ROX+) (AS-MR-006-5, Arraystar), and snoRNA profiling was performed by using nrStar™ Human snoRNA PCR array according to the manufacturer's instructions (Arraystar, Inc, US) on ABI 7900 real-time PCR system (Applied Biosystems, Foster City, CA). The PCR array consisted of 384 primer sets for analyzing small nucleolar RNAs.

### 2.4. Verification of SNORD60 Expression by Quantitative RT-PCR

The expression levels of SNORD60 were verified using 12 pair-matched surgically resected LUAD tissues. Total RNA was extracted according to the manufacturer's procedure using an RNA extraction kit (Cat# R1200, Suolaibao, China). General reverse transcription kit (Cat# 11141ES60, Yisheng Biology, China) was used to make cDNA. Fluorescence quantification kit (Cat# 11201ES08, Yisheng Biology, China) was used to perform real-time PCR. As an endogenous control, the U6 gene was employed. The upstream and downstream amplification sequences are listed in Table S3 in the Supplementary Material.

## 3. Statistical Analysis

The statistical analyses were performed using R (v4.1.2) [15, 16]. Differentially expressed snoRNAs were detected by R packages “edgeR” and “limma.” The diagnostic ability of SONRD60 was assessed using a receiver operating characteristic (ROC) curve by R package “pROC” [17]. The correlation between SNORD60 expression and clinicopathological features was assessed using the Wilcoxon test. The Kaplan-Meier survival analysis was performed by log-rank test using “survival” R package [18]. Univariate and subsequent multivariate Cox regression analyses were used to determine the independent prognostic significance of SNORD60 expression on LUAD. The threshold for statistical significance was fixed at *P* < 0.05.

## 4. Results

### 4.1. SNORD60 Was Overexpressed in LUAD

**Figure 1:**
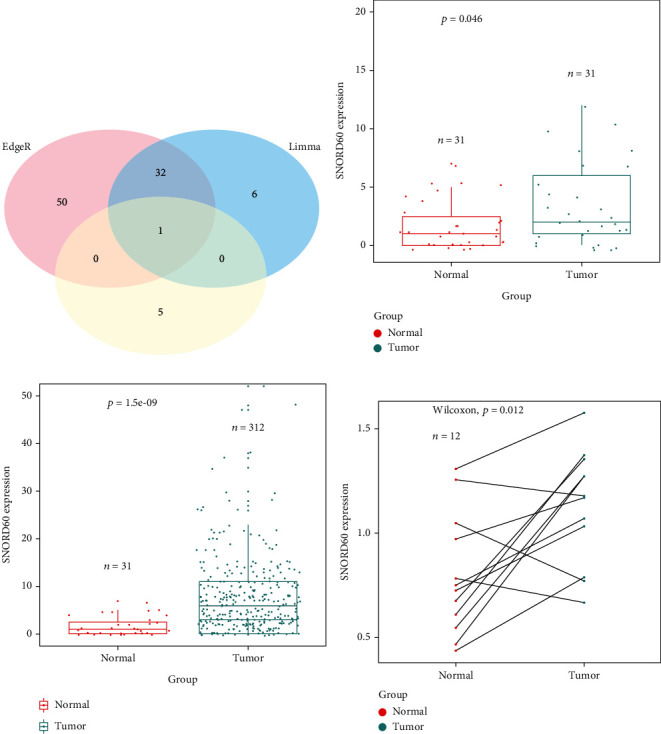
SNORD60 expression status in LUAD. (a) The intersection analysis of upregulated snoRNA profiling. (b, c) TCGA cohort showed that SNORD60 expression levels were significantly higher in paired and unpaired LUAD tissues (*P* < 0.05). (d) qRT-PCR-verified SNORD60 was significantly overexpressed in 12 pair-matched LUAD samples (*P* < 0.05), expression normalized to U6.

**Figure 2:**
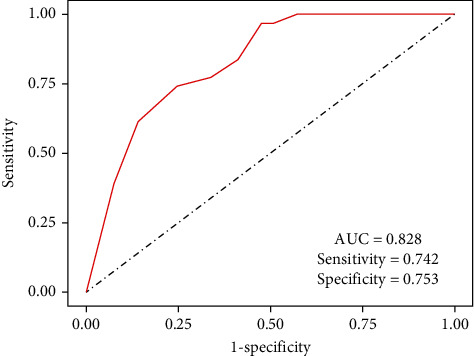
Receiver operating characteristic (ROC) curve analysis indicated that SNORD60 expression levels distinguished LUAD tissues from matched adjacent normal tissues.

By examining TCGA database using “edgeR” (|Log FC |>0.585, adjusted *P* < 0.05, Supplementary Table S4), a total of 97 substantially differential expression snoRNAs were discovered in tumors compared with normal tissues, comprising 83 upregulated and 14 downregulated snoRNAs. Meanwhile, using “limma” (|Log FC|>0.585, adjusted *P* < 0.05, Supplementary Table S5), 63 highly differentially expressed snoRNAs were identified, comprising 39 upregulated and 24 downregulated snoRNAs. The snoRNA PCR array revealed that five upregulated and five downregulated snoRNAs were differently expressed in three surgically resected LUAD tissues compared to normal tissues (fold difference > 1.5, *P* < 0.05, Supplementary Table S6). The intersection of these three datasets yielded only one upregulated snoRNA (SNORD60) (Figure 1(a)). SNORD60 expression was determined using the LUAD TCGA dataset, which revealed that SNORD60 expression levels were significantly higher in paired (*n* = 31) and unpaired (*n* = 312) LUAD tissues (Figures 1(b)–1(c)). SNORD60 expression was verified using quantitative RT-PCR in 12 pair-matched surgically resected LUAD tissues, which showed that LUAD tissues had considerably higher SNORD60 expression levels than noncancerous lung tissues (*P* < 0.05) (Figure 1(d)). Furthermore, the ROC curve analysis revealed that SNORD60 expression could distinguish between LUAD and normal cases (area under the curve: 0.828, sensitivity: 0.742, and specificity: 0.753), implying that SNORD60 could be used as a potential diagnostic biomarker for LUAD patients (Figure 2).

### 4.2. The Expression of SNORD60 and Its Relationship to Clinicopathological Factors

**Figure 3:**
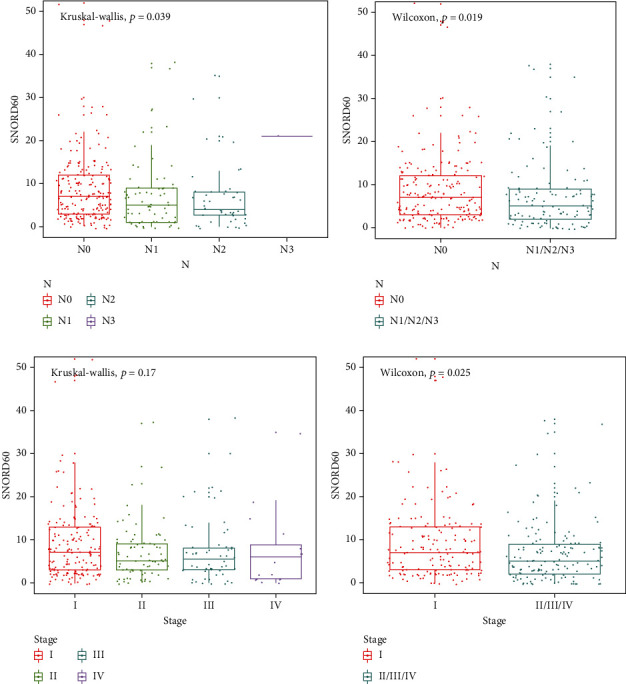
The association between SNORD60 expression and clinicopathological characteristics. (a, b) SNORD60 expression was associated with lymph node metastases (*P* < 0.05). (c, d) SNORD60 expression was associated with TNM stage (I vs. II/III/IV, *P* < 0.05).

The association between SNORD60 expression and clinicopathological characteristics, including sex, age, TNM stage, pathological T category, lymph node metastasis, and distant metastasis, was investigated. As shown in Figure 3, SNORD60 expression was significantly linked with lymph node metastases and TNM stage (*P* < 0.05) but displayed no correlation with other clinical parameters (*P* > 0.05, Supplementary Figure S2). Patients with early-stage LUAD who do not have regional lymph node metastases are more likely to have increased SNORD60 expression. Furthermore, LUAD patients were divided into high- and low-expression groups according to SNORD60 expression levels (median value). The Kaplan-Meier analysis revealed no significant differences in overall survival between the two groups (*P* > 0.05, Supplementary Figure S3).

### 4.3. The Association between SNORD60 and Prognosis

**Figure 4:**
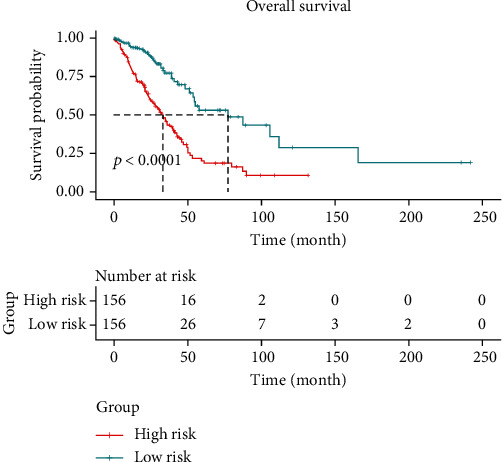
Kaplan-Meier analysis of the prognostic model. The LUAD patients with high risk exhibited worse survival.

To further investigate the prognostic value of SNORD60, univariate and multivariate Cox regression was conducted to analyze the prognostic factors that affect the overall survival of patients with LUAD. Multiple variables (TNM stage, pathological T category, lymph node metastasis, and distant metastasis) were shown to be substantially associated with overall survival in univariate analysis, as indicated in Table 1. In multivariate analysis, only pathological T category and lymph node metastases were shown to be independent prognostic markers for LUAD overall survival. The prognostic model risk score's Kaplan-Meier survival curve demonstrated that high-risk patients had a lower survival rate (*P* < 0.0001, Figure 4).

## 5. Discussion

LUAD is the most prevalent subtype with the greatest incidence among lung cancer patients in China. Unlike lung squamous cell carcinoma (LUSC), LUAD is more frequent in women and nonsmokers and arises primarily from the bronchial mucosal epithelium, with no early clinical signs. As a result, many LUAD patients with poor prognoses were detected in the middle and late stages [1, 2]. With breakthroughs in finding novel biomarkers, our understanding of the etiology of LUAD has increased [19–21].

According to a growing body of data, snoRNAs have a crucial role in the genesis of cancer [22, 23]. The study of snoRNAs in LUAD might be used as clinical biomarkers and therapeutic targets. According to genome-wide research, RNAs are encoded from around 80% of the human genome, but only 1.5 percent of these encoded RNAs can be translated into proteins [24]. As a result, most of the RNA in cells is noncoding RNA (ncRNA). snoRNAs are 60–300-nucleotide long members of the ncRNA family found in the nucleolus of eukaryotic cells [6]. snoRNAs are involved in the processing and editing of different RNAs as a guide for RNA-dependent RNA modification and form snoRNP complexes with nucleoli ribosomal proteins [25–27]. snoRNAs are classified into four groups based on their structure and function: box C/D snoRNAs (SNORDs), box H/ACA snoRNAs (SNORAs), small Cajal RNA (scaRNA), and orphan snoRNAs. Box C/D and box H/ACA are the best-known snoRNA types, which primarily guide the hydroxymethylation and pseudouracil modification of ribosomal RNA through base pairings [28].

To eliminate the impact of neoadjuvant therapy variables on snoRNA expression level, we defined rigid screening criteria for TCGA database data in advance. The LUAD TCGA database and pair-matched surgical LUAD tissues were used to find snoRNAs with differential expression. After crossing the analysis data, only one significant upregulated snoRNA, SNORD60, was discovered. SNORD60 expression distinguished LUAD patients from normal cases, according to receiver operating characteristic studies. Even though the survival curves for high and low SNORD60 expression groups were negative (*P* = 0.46), which could be due to a large number of patients in stage I (*n* = 164, 52.6%), increased SNORD60 expression was significantly associated with early stage, indicating that SNORD60 can be used for LUAD early diagnosis. All the above findings suggested that aberrant SNORD60 expression might be an oncogenic factor.

Recent evidence showed that snoRNAs are mostly overexpressed in cancer tissues relative to normal samples and that their biological activities play a role in cancer formation and oncogenesis. Using small RNA sequencing, Zhang et al. discovered that SNORA71A was highly elevated in colorectal cancer (CRC) tissues and confirmed by RT-qPCR and TCGA data analysis. SNORA71A served as an oncogene in subsequent functional studies, promoting CRC cell proliferation, motility, and invasion ability [29]. SNORD16 was shown to be overexpressed in colon cancer (CC) tissues and to be inversely linked with overall survival in CC patients, according to He et al. High SNORD16 expression was found to be an independent predictive factor for CC in a multivariate Cox proportional hazards model [30]. Li et al. discovered that elevated SNHG6 expression was linked with pathological stage and lymph node infiltration, serving as an independent predictive predictor of tumor recurrence in NSCLC patients. By sponging miR-101-3p, lncRNA SNHG6 promoted cell proliferation and invasion in NSCLC cells [31]. Based on TCGA dataset, Zhang et al. discovered a novel predictive expression profile of snoRNAs (snoU109, SNORA5A, SNORA70, SNORD104, and U3) associated with lung adenocarcinoma. Biological functional investigation revealed that LUAD patients with varying risk score characteristics showed substantial variations in several signaling pathways [11].

Our investigation found that SNORD60 plays a role in LUAD carcinogenesis and development and might be a potential prognostic biomarker. Zou et al. used TCGA database to extract RNA-sequencing expression data from 31 head and neck squamous cell carcinoma (HNSCC) and pair-matched normal tissues, followed by “edgeR” to examine dysregulated snoRNAs. A total of 33 differently expressed snoRNAs were identified, including SNORD60, which was upregulated (superior to 2 folds) in HNSCC [32]. SNORD60, also known as U60/rNU60, is encoded by an 83 bp genome on chromosome 16p13.3, the most commonly amplified chromosomal regions which may have an oncogenic role in developing different cancers [33–35]. The long noncoding RNA- (lncRNA-) SNHG19 is also found on the same chromosome. Li et al. discovered a panel of lncRNAs, including SNHG19, which are significantly expressed in breast cancer and may be utilized as a predictor of survival [36].

## 6. Conclusion

Our findings demonstrated that SNORD60, a small nucleolar RNA, has an oncogenic function in LUAD and might be used as a new diagnostic biomarker for LUAD. Due to the limited sample size in TCGA official website, our findings still require confirmation, and the biological functioning processes must be tested in vivo and in vitro. Future experiments are aimed at investigating the biological significance of SNORD60 in carcinogenesis. The discovery of snoRNAs in lung cancer may open the door for novel therapeutic applications.

## Figures and Tables

**Table 1:** Cox regression for overall survival analysis.

Variables	Univariate cox		Multivariate cox	
	HR (95% CI)	*P* value	HR (95% CI)	*P* value
Age (years)				
≥65	1			
<65	0.872 (0.614-1.238)	0.444		
Gender				
Female	1			
Male	1.046 (0.739-1.481)	0.798		
Stage				
I	1			
II	2.159 (1.386-3.363)	0.001^a^		
III	3.253 (2.083-5.080)	≤0.001^a^		
IV	3.145 (1.692-5.844)	≤0.001^a^		
T				
T1	1			
T2	1.789 (1.122-2.853)	0.015^a^	1.590 (0.995-2.543)	0.053
T3	4.726 (2.505-8.917)	≤0.001^a^	4.151 (2.150-8.014)	≤0.001^a^
T4	3.133 (1.522-6.452)	0.002^a^	2.149 (1.001-4.613)	0.050
N				
N0	1			
N1	2.293 (1.519-3.463)	≤0.001^a^	2.220 (1.457-3.381)	≤0.001^a^
N2	3.209 (2.073-4.970)	≤0.001^a^	2.584 (1.641-4.070)	≤0.001^a^
N3	3.795e-07 (0.000-inf)	0.995	2.360e-07 (0.000-inf)	0.995
M				
M0	1			
M1	1.965 (1.104-3.495)	0.022^a^	1.708 (0.927-3.148)	0.086
SNORD60_group^b^				
High	1			
Low	1.140 (0.802-1.621)	0.465		

Abbreviation: CI: confidence interval; inf: infinity. ^a^*P* < 0.05. ^b^Cutoff threshold of SNORD60 expression is median value in all patients.

## Data Availability

The datasets used and analyzed during the current study are available from the corresponding author on reasonable request.

## Supplementary Materials

Supplementary Materials Supplementary Table S1: the demographic and clinicopathological characteristics of TCGA LUAD cohort. Supplementary Table S2: the clinicopathologic characteristics of LUAD patients. Supplementary Table S3: amplification sequences in quantitative RT-PCR. Supplementary Table S4: differential expression snoRNAs of LUAD TCGA database were detected by R packages “edgeR”. Supplementary Table S5: differential expression snoRNAs of LUAD TCGA database were detected by R packages “limma.” Supplementary Table S6: snoRNA profiling in three matched surgically resected LUAD tissues. Supplementary Figure S1: a screenshot of the file filter settings in TCGA website (https://portal.gdc.cancer.gov). Supplementary Figure S2: the relationship between SNORD60 expression and other clinical factors (including age, sex, pathological T category, and distant metastasis). Supplementary Figure S3: Kaplan-Meier analysis of overall survival. There was no difference between the high and low SNORD60 expression groups (*P* > 0.05). The median expression level of SNORD60 was used as the cutoff.

## References

[1] Sung H., Ferlay J., Siegel R. L. (2021). Global cancer statistics 2020: GLOBOCAN estimates of incidence and mortality worldwide for 36 cancers in 185 countries. *CA: a Cancer Journal for Clinicians*.

[2] Yang D., Liu Y., Bai C., Wang X., Powell C. A. (2020). Epidemiology of lung cancer and lung cancer screening programs in China and the United States. *Cancer Letters*.

[3] Travis W. D., Brambilla E., Nicholson A. G. (2015). The 2015 World Health Organization classification of lung tumors: impact of genetic, clinical and radiologic advances since the 2004 classification. *Journal of Thoracic Oncology: Official Publication of the International Association for the Study of Lung Cancer*.

[4] Wang Q., Li M., Yang M. (2020). Analysis of immune-related signatures of lung adenocarcinoma identified two distinct subtypes: implications for immune checkpoint blockade therapy. *Aging*.

[5] Zengin T., Önal-Süzek T. (2020). Analysis of genomic and transcriptomic variations as prognostic signature for lung adenocarcinoma. *BMC bioinformatics*.

[6] Bratkovič T., Božič J., Rogelj B. (2020). Functional diversity of small nucleolar RNAs. *Nucleic Acids Research*.

[7] Gong J., Li Y., Liu C. J. (2017). A pan-cancer analysis of the expression and clinical relevance of small nucleolar RNAs in human cancer. *Cell Reports*.

[8] Liang J., Wen J., Huang Z., Chen X. P., Zhang B. X., Chu L. (2019). Small nucleolar RNAs: insight into their function in cancer. *Frontiers in oncology*.

[9] Liao J., Yu L., Mei Y. (2010). Small nucleolar RNA signatures as biomarkers for non-small-cell lung cancer. *Molecular cancer*.

[10] Zhang Z., Zhang J., Diao L., Han L. (2021). Small non-coding RNAs in human cancer: function, clinical utility, and characterization. *Oncogene*.

[11] Zhang L., Xin M., Wang P. (2021). Identification of a novel snoRNA expression signature associated with overall survival in patients with lung adenocarcinoma: a comprehensive analysis based on RNA sequencing dataset. *Mathematical biosciences and engineering: MBE*.

[12] Collisson E. A. C. J., Campbell J., Brooks A. (2014). Comprehensive molecular profiling of lung adenocarcinoma. *Nature*.

[13] Petersen I., Warth A. (2016). Lung cancer: developments, concepts, and specific aspects of the new WHO classification. *Journal of Cancer Research and Clinical Oncology*.

[14] Osmani L., Askin F., Gabrielson E., Li Q. K. (2018). Current WHO guidelines and the critical role of immunohistochemical markers in the subclassification of non-small cell lung carcinoma (NSCLC): moving from targeted therapy to immunotherapy. *Seminars in Cancer Biology*.

[15] Team R. C. (2008). R A language and environment for statistical computing 2014. *R Foundation for Statistical Computing*.

[16] Ginestet C. (2011). ggplot2: elegant graphics for data analysis. *Journal of the Royal Statistical Society*.

[17] Robin X., Turck N., Hainard A. (2011). pROC: an open-source package for R and S+ to analyze and compare ROC curves. *BMC bioinformatics*.

[18] Melnick E. L. (2011). *Modeling Survival Data*.

[19] Liu X. X., Yang Y. E., Liu X. (2019). A two-circular RNA signature as a noninvasive diagnostic biomarker for lung adenocarcinoma. *Journal of translational medicine*.

[20] Inamura K. (2018). Clinicopathological characteristics and mutations driving development of early lung adenocarcinoma: tumor initiation and progression. *International journal of molecular sciences*.

[21] Chen Y. J., Roumeliotis T. I., Chang Y. H. (2020). Proteogenomics of non-smoking lung cancer in East Asia delineates molecular signatures of pathogenesis and progression. *Cell*.

[22] Romano G., Veneziano D., Acunzo M., Croce C. M. (2017). Small non-coding RNA and cancer. *Carcinogenesis*.

[23] Wajahat M., Bracken C. P., Orang A. (2021). Emerging functions for snoRNAs and snoRNA-derived fragments. *International journal of molecular sciences*.

[24] Mourksi N. E., Morin C., Fenouil T., Diaz J. J., Marcel V. (2020). snoRNAs offer novel insight and promising perspectives for lung cancer understanding and management. *Cells*.

[25] Sloan K. E., Warda A. S., Sharma S., Entian K. D., Lafontaine D., Bohnsack M. T. (2017). Tuning the ribosome: the influence of rRNA modification on eukaryotic ribosome biogenesis and function. *RNA Biology*.

[26] Zhao Y., Yan Y., Ma R. (2020). Expression signature of six-snoRNA serves as novel non-invasive biomarker for diagnosis and prognosis prediction of renal clear cell carcinoma. *Journal of Cellular and Molecular Medicine*.

[27] Cui C., Liu Y., Gerloff D. (2021). NOP10 predicts lung cancer prognosis and its associated small nucleolar RNAs drive proliferation and migration. *Oncogene*.

[28] Dupuis-Sandoval F., Poirier M., Scott M. S. (2015). The emerging landscape of small nucleolar RNAs in cell biology. *Wiley interdisciplinary reviews. RNA*.

[29] Zhang Z., Tao Y., Hua Q., Cai J., Ye X., Li H. (2020). SNORA71A promotes colorectal cancer cell proliferation, migration, and invasion. *BioMed research international*.

[30] He J.-y., Liu X., Qi Z.-h. (2020). Small nucleolar RNA, C/D box 16 (SNORD16) acts as a potential prognostic biomarker in colon cancer. *Dose-response: a publication of International Hormesis Society*.

[31] Li K., Jiang Y., Xiang X. (2020). Long non-coding RNA SNHG6 promotes the growth and invasion of non-small cell lung cancer by downregulating miR-101-3p. *Thoracic cancer*.

[32] Zou A. E., Ku J., Honda T. K. (2015). Transcriptome sequencing uncovers novel long noncoding and small nucleolar RNAs dysregulated in head and neck squamous cell carcinoma. *RNA*.

[33] Bramhecha Y. M., Guérard K. P., Rouzbeh S. (2018). Genomic gain of 16p13.3 in prostate cancer predicts poor clinical outcome after surgical intervention. *Molecular cancer research: MCR*.

[34] Mampaey E., Fieuw A., Van Laethem T. (2015). Focus on 16p13.3 locus in colon cancer. *PloS one*.

[35] Choucair K. A., Guérard K. P., Ejdelman J. (2012). The 16p13.3 (PDPK1) genomic gain in prostate cancer: a potential role in disease progression. *Translational Oncology*.

[36] Li X.-X., Wang L.-J., Hou J. (2020). Identification of long noncoding RNAs as predictors of survival in triple-negative breast cancer based on network analysis. *BioMed research international*.

