# Supplementary material for: Identification of Small Nucleolar RNA SNORD60 as a Potential Biomarker and Its Clinical Significance in Lung Adenocarcinoma
Source: Biomed Res Int. 2022 Jun 7;2022:5501171. doi: 10.1155/2022/5501171 (PMC9197630; doi:10.1155/2022/5501171)
Supplement: Supplementary Materials — Supplementary Table S1: the demographic and clinicopathological characteristics of TCGA LUAD cohort. Supplementary Table S2: the clinicopathologic characteristics of LUAD patients. Supplementary Table S3: amplification sequences in quantitative RT-PCR. Supplementary Table S4: differential expression snoRNAs of LUAD TCGA database were detected by R packages “edgeR”. Supplementary Table S5: differential expression snoRNAs of LUAD TCGA database were detected by R packages “limma.” Supplementary Table S6: snoRNA profiling in three matched surgically resected LUAD tissues. Supplementary Figure S1: a screenshot of the file filter settings in TCGA website (https://portal.gdc.cancer.gov). Supplementary Figure S2: the relationship between SNORD60 expression and other clinical factors (including age, sex, pathological T category, and distant metastasis). Supplementary Figure S3: Kaplan-Meier analysis of overall survival. There was no difference between the high and low SNORD60 expression groups (P > 0.05). The median expression level of SNORD60 was used as the cutoff. [file 5501171.f1.zip › table S3.docx]

Table S3. Amplification sequences in Quantitative RT-PCR

|  | primer sequences | | BP |
| --- | --- | --- | --- |
| U6 | F | CTCGCTTCGGCAGCACA | 17 |
|  | R | AACGCTTCACGAATTTGCGT | 20 |
| SNORD60 | F | TTGCTTTGACTTCTGACACCTCGTA | 25 |
|  | R | GTCTTGCTAAATAATCAGACTGCACG | 26 |

Ps. The annealing temperature: 60℃; GC content of prime: 43.1%
