# Supplementary material for: Identification of Small Nucleolar RNA SNORD60 as a Potential Biomarker and Its Clinical Significance in Lung Adenocarcinoma
Source: Biomed Res Int. 2022 Jun 7;2022:5501171. doi: 10.1155/2022/5501171 (PMC9197630; doi:10.1155/2022/5501171)
Supplement: Supplementary Materials — Supplementary Table S1: the demographic and clinicopathological characteristics of TCGA LUAD cohort. Supplementary Table S2: the clinicopathologic characteristics of LUAD patients. Supplementary Table S3: amplification sequences in quantitative RT-PCR. Supplementary Table S4: differential expression snoRNAs of LUAD TCGA database were detected by R packages “edgeR”. Supplementary Table S5: differential expression snoRNAs of LUAD TCGA database were detected by R packages “limma.” Supplementary Table S6: snoRNA profiling in three matched surgically resected LUAD tissues. Supplementary Figure S1: a screenshot of the file filter settings in TCGA website (https://portal.gdc.cancer.gov). Supplementary Figure S2: the relationship between SNORD60 expression and other clinical factors (including age, sex, pathological T category, and distant metastasis). Supplementary Figure S3: Kaplan-Meier analysis of overall survival. There was no difference between the high and low SNORD60 expression groups (P > 0.05). The median expression level of SNORD60 was used as the cutoff. [file 5501171.f1.zip › table S4.doc]

Table S4. Differential expression snoRNAs of LUAD TCGA database were detected by R packages “edgeR”

| **Name** | **logFC** | ***P*-value** | **FDR** | **change** |
| --- | --- | --- | --- | --- |
| SNORA26 | 1.800170238 | 2.27E-20 | 5.97E-18 | UP |
| SNORD99 | 1.520485089 | 7.32E-20 | 1.44E-17 | UP |
| SNORA73B | 3.12292339 | 7.58E-14 | 8.54E-12 | UP |
| SNORD60 | 1.578084355 | 1.01E-13 | 1.00E-11 | UP |
| SNORD7 | 1.494935955 | 8.08E-13 | 7.08E-11 | UP |
| SNORD46 | 1.393848675 | 2.20E-12 | 1.73E-10 | UP |
| SNORA14B | 1.841314544 | 2.15E-11 | 1.38E-09 | UP |
| SNORD14E | 1.713861555 | 2.23E-11 | 1.38E-09 | UP |
| SNORD19C | 1.124975523 | 2.27E-11 | 1.38E-09 | UP |
| SNORD104 | 1.081597764 | 8.51E-10 | 4.20E-08 | UP |
| SNORA71A | 1.637954108 | 1.01E-09 | 4.71E-08 | UP |
| SNORA71C | 1.295419399 | 2.17E-09 | 9.00E-08 | UP |
| SNORD17 | 2.137517404 | 3.65E-09 | 1.44E-07 | UP |
| SNORD72 | 1.405441087 | 6.64E-09 | 2.50E-07 | UP |
| SNORA71B | 0.92119949 | 9.70E-09 | 3.48E-07 | UP |
| ENSG10010139025.1 | 2.086966206 | 1.42E-08 | 4.87E-07 | UP |
| SNORD124 | 1.581345761 | 1.64E-08 | 5.38E-07 | UP |
| SNORA38B | 1.999175232 | 2.38E-08 | 7.52E-07 | UP |
| SCARNA12 | 1.559957548 | 5.70E-08 | 1.73E-06 | UP |
| ENSG10010137428.1 | 2.488264816 | 9.35E-08 | 2.73E-06 | UP |
| SNORA60 | 0.892618336 | 1.36E-07 | 3.82E-06 | UP |
| ENSG10010138531.1 | 2.314324782 | 1.41E-07 | 3.84E-06 | UP |
| SNORA71D | 3.643356008 | 1.83E-07 | 4.81E-06 | UP |
| SNORA23 | 2.796926398 | 2.29E-07 | 5.83E-06 | UP |
| SNORA80E | 1.331101214 | 2.40E-07 | 5.91E-06 | UP |
| SNORD12B | 0.905448165 | 2.52E-07 | 6.03E-06 | UP |
| SNORD93 | 1.57638842 | 3.76E-07 | 8.72E-06 | UP |
| SNORA5C | 0.789349561 | 6.36E-07 | 1.43267E-05 | UP |
| ENSG10010138347.1 | 2.098101417 | 9.40E-07 | 2.0602E-05 | UP |
| SNORD94 | 0.796991968 | 1.35E-06 | 2.88802E-05 | UP |
| ENSG10010137705.1 | 2.404825724 | 2.92E-06 | 6.06517E-05 | UP |
| SNORA69 | 1.178297789 | 3.47E-06 | 7.0249E-05 | UP |
| SNORD117 | 1.053487271 | 4.95E-06 | 9.76093E-05 | UP |
| SNORA72 | 1.21759437 | 6.69E-06 | 0.00012871 | UP |
| SNORD15B | 1.294150253 | 9.90E-06 | 0.000185962 | UP |
| SNORA5A | 0.958312905 | 1.02606E-05 | 0.000188269 | UP |
| SCARNA10 | 2.363326565 | 1.18232E-05 | 0.000212011 | UP |
| SNORA53 | 1.453074784 | 1.48287E-05 | 0.000254344 | UP |
| SNORA80A | 2.731532718 | 3.97041E-05 | 0.000666522 | UP |
| ENSG10010135192.1 | 2.218275373 | 4.57833E-05 | 0.000752563 | UP |
| SNORA11 | 0.821233896 | 5.28781E-05 | 0.000851445 | UP |
| ENSG10010138890.1 | 0.693933771 | 7.77461E-05 | 0.001202777 | UP |
| SNORD69 | 0.730283686 | 9.55701E-05 | 0.001448217 | UP |
| SNORA79B | 1.549182941 | 9.7282E-05 | 0.001448217 | UP |
| ENSG10010134634.1 | 1.681802874 | 0.000171462 | 0.0024597 | UP |
| SNORD67 | 1.316301588 | 0.000191337 | 0.002695806 | UP |
| SNORA74D | 2.397583525 | 0.000243113 | 0.00330717 | UP |
| SNORA74B | 1.588045346 | 0.000280495 | 0.003751025 | UP |
| SNORD53 | 1.647681722 | 0.000329406 | 0.004331686 | UP |
| SNORD12C | 0.935736607 | 0.00034285 | 0.004434562 | UP |
| SNORA84 | 2.588191447 | 0.000351041 | 0.004467285 | UP |
| ENSG10010139899.1 | 0.698759545 | 0.000392477 | 0.004915305 | UP |
| SNORA80B | 1.365372311 | 0.000486297 | 0.005813459 | UP |
| SNORA74A | 2.868368694 | 0.000500237 | 0.005890854 | UP |
| SNORA63D | 2.168680221 | 0.000529603 | 0.006055894 | UP |
| SNORD70 | 1.395513054 | 0.000540729 | 0.006094793 | UP |
| SNORA79 | 1.639587897 | 0.00064839 | 0.007186537 | UP |
| ENSG10010136707.1 | 1.399615276 | 0.000655806 | 0.007186537 | UP |
| SNORA58B | 1.018701492 | 0.000801511 | 0.008545837 | UP |
| SNORA49 | 2.518448773 | 0.000830702 | 0.008738988 | UP |
| SNORD88A | 1.085523138 | 0.000899354 | 0.009215454 | UP |
| SNORA40B | 1.945063138 | 0.001040849 | 0.010279763 | UP |
| SNORA54 | 2.112830526 | 0.001042308 | 0.010279763 | UP |
| ENSG10010137899.1 | 1.624681383 | 0.001201642 | 0.011704886 | UP |
| ENSG10010138241.1 | 1.090061705 | 0.001238056 | 0.011912519 | UP |
| SNORA9B | 0.700715887 | 0.001305882 | 0.012413743 | UP |
| SNORD19 | 0.622445284 | 0.001545576 | 0.014517373 | UP |
| SCARNA18B | 1.379196189 | 0.001575117 | 0.014620788 | UP |
| SNORA12 | 1.202097196 | 0.001823983 | 0.016733981 | UP |
| SNORD15A | 1.011550631 | 0.001862087 | 0.016887206 | UP |
| snoZ196 | 0.626388173 | 0.002090231 | 0.018740817 | UP |
| SNORD48 | 0.96090414 | 0.00240448 | 0.021316123 | UP |
| ENSG10010137195.1 | 2.766378106 | 0.002471182 | 0.02166403 | UP |
| SNORA14A | 1.240166895 | 0.003152914 | 0.026748919 | UP |
| SNORA80C | 1.367674478 | 0.003566767 | 0.029938073 | UP |
| SNORA2C | 0.79705266 | 0.003606153 | 0.029950053 | UP |
| ENSG10010135413.1 | 1.229489071 | 0.004277909 | 0.034796603 | UP |
| SNORD73B | 0.625329854 | 0.004819889 | 0.038805021 | UP |
| ENSG10010139579.1 | 0.594068851 | 0.00491171 | 0.03914484 | UP |
| ENSG10010135973.1 | 1.369780841 | 0.005169578 | 0.04078797 | UP |
| SNORA22C | 1.15758592 | 0.005826202 | 0.045513595 | UP |
| ENSG10010135706.1 | 1.338675811 | 0.005909833 | 0.045714296 | UP |
| SNORD11B | 1.101593941 | 0.006150766 | 0.047116063 | UP |
| ENSG10010134187.1 | -2.191055105 | 1.07E-25 | 8.43E-23 | DOWN |
| ENSG10010134689.1 | -2.111393494 | 4.96E-25 | 1.96E-22 | DOWN |
| ENSG10010135879.1 | -1.733561947 | 4.01E-17 | 6.32E-15 | DOWN |
| ENSG10010136194.1 | -1.418049894 | 4.37E-15 | 5.74E-13 | DOWN |
| ENSG10010137877.1 | -2.371776859 | 3.78E-11 | 2.13E-09 | DOWN |
| ENSG10010136902.1 | -1.036826594 | 1.56E-10 | 8.22E-09 | DOWN |
| ENSG10010136633.1 | -2.113646482 | 1.30E-09 | 5.69E-08 | DOWN |
| SNORA77 | -0.807997904 | 1.34339E-05 | 0.000235541 | DOWN |
| ENSG10010137260.1 | -1.958292423 | 0.000165778 | 0.002422194 | DOWN |
| SNORD116-4 | -0.867613792 | 0.000233546 | 0.003232773 | DOWN |
| ENSG10010136964.1 | -0.986882936 | 0.000437792 | 0.005375569 | DOWN |
| ENSG10010136497.1 | -1.119207575 | 0.000513047 | 0.005952854 | DOWN |
| SNORA70B | -0.951452735 | 0.000729793 | 0.007887766 | DOWN |
| ENSG10010135283.1 | -0.776794808 | 0.001007132 | 0.010187525 | DOWN |
