# Supplementary material for: Identification of Small Nucleolar RNA SNORD60 as a Potential Biomarker and Its Clinical Significance in Lung Adenocarcinoma
Source: Biomed Res Int. 2022 Jun 7;2022:5501171. doi: 10.1155/2022/5501171 (PMC9197630; doi:10.1155/2022/5501171)
Supplement: Supplementary Materials — Supplementary Table S1: the demographic and clinicopathological characteristics of TCGA LUAD cohort. Supplementary Table S2: the clinicopathologic characteristics of LUAD patients. Supplementary Table S3: amplification sequences in quantitative RT-PCR. Supplementary Table S4: differential expression snoRNAs of LUAD TCGA database were detected by R packages “edgeR”. Supplementary Table S5: differential expression snoRNAs of LUAD TCGA database were detected by R packages “limma.” Supplementary Table S6: snoRNA profiling in three matched surgically resected LUAD tissues. Supplementary Figure S1: a screenshot of the file filter settings in TCGA website (https://portal.gdc.cancer.gov). Supplementary Figure S2: the relationship between SNORD60 expression and other clinical factors (including age, sex, pathological T category, and distant metastasis). Supplementary Figure S3: Kaplan-Meier analysis of overall survival. There was no difference between the high and low SNORD60 expression groups (P > 0.05). The median expression level of SNORD60 was used as the cutoff. [file 5501171.f1.zip › tableS1 (1).doc]

Table S1. The demographic and clinicopathological characteristics of the TCGA LUAD cohort

| Characteristics | Number (%) |
| --- | --- |
| Age (years) |  |
| ＜65 | 159（51.0%） |
| ≥65 | 153（49.0%） |
| Gender |  |
| Female | 161（51.6%） |
| Male | 151（48.4%） |
| Stage |  |
| I | 164（52.6%） |
| II | 74（23.7%） |
| III | 54（17.3%） |
| IV | 20（6.4%） |
| T |  |
| T1 | 96（30.8%） |
| T2 | 174（55.8%） |
| T3 | 25（8.0%） |
| T4 | 17（5.4%） |
| N |  |
| N0 | 199（63.8%） |
| N1 | 64（20.5%） |
| N2 | 48（15.4%） |
| N3 | 1（0.3%） |
| M |  |
| M0 | 292（93.6%） |
| M1 | 20（6.4%） |
