# Supplementary material for: Identification of Small Nucleolar RNA SNORD60 as a Potential Biomarker and Its Clinical Significance in Lung Adenocarcinoma
Source: Biomed Res Int. 2022 Jun 7;2022:5501171. doi: 10.1155/2022/5501171 (PMC9197630; doi:10.1155/2022/5501171)
Supplement: Supplementary Materials — Supplementary Table S1: the demographic and clinicopathological characteristics of TCGA LUAD cohort. Supplementary Table S2: the clinicopathologic characteristics of LUAD patients. Supplementary Table S3: amplification sequences in quantitative RT-PCR. Supplementary Table S4: differential expression snoRNAs of LUAD TCGA database were detected by R packages “edgeR”. Supplementary Table S5: differential expression snoRNAs of LUAD TCGA database were detected by R packages “limma.” Supplementary Table S6: snoRNA profiling in three matched surgically resected LUAD tissues. Supplementary Figure S1: a screenshot of the file filter settings in TCGA website (https://portal.gdc.cancer.gov). Supplementary Figure S2: the relationship between SNORD60 expression and other clinical factors (including age, sex, pathological T category, and distant metastasis). Supplementary Figure S3: Kaplan-Meier analysis of overall survival. There was no difference between the high and low SNORD60 expression groups (P > 0.05). The median expression level of SNORD60 was used as the cutoff. [file 5501171.f1.zip › tableS5.doc]

Table S5. Differential expression snoRNAs of LUAD TCGA database were detected by R packages “limma”

| **Name** | **logFC** | ***P*-value** | **FDR** | **change** |
| --- | --- | --- | --- | --- |
| SNORD7 | 2.301762422 | 4.59E-49 | 3.62E-46 | UP |
| SNORD117 | 1.779822062 | 2.38E-41 | 9.39E-39 | UP |
| SNORD73B | 1.320358749 | 4.19E-41 | 1.10E-38 | UP |
| SNORA80B | 1.792367523 | 2.13E-32 | 3.36E-30 | UP |
| ENSG10010137428.1 | 0.941240014 | 4.87E-23 | 4.80E-21 | UP |
| SNORA26 | 1.706023999 | 3.61E-20 | 3.04E-18 | UP |
| SNORA80E | 0.997627059 | 7.86E-20 | 5.64E-18 | UP |
| ENSG10010136469.1 | 0.910284735 | 1.67E-15 | 1.09E-13 | UP |
| SNORD99 | 1.362683665 | 3.40E-14 | 1.92E-12 | UP |
| SNORA71D | 0.660732903 | 5.50E-14 | 2.71E-12 | UP |
| SNORA22C | 0.854785748 | 7.49E-14 | 3.48E-12 | UP |
| SNORA73B | 0.960616954 | 1.50E-12 | 5.65E-11 | UP |
| SNORD46 | 1.207421027 | 1.48E-12 | 5.65E-11 | UP |
| SNORA46 | 0.883642963 | 1.91E-12 | 6.85E-11 | UP |
| SNORA71A | 1.232685132 | 3.34E-11 | 1.12E-09 | UP |
| ENSG10010139579.1 | 1.14549262 | 6.19E-11 | 1.95E-09 | UP |
| SNORD91B | 0.921331431 | 1.02E-10 | 2.98E-09 | UP |
| SNORD3A | 0.928377611 | 4.24E-10 | 1.20E-08 | UP |
| ENSG10010138241.1 | 0.841966833 | 6.81E-10 | 1.79E-08 | UP |
| SNORD19C | 1.063836507 | 5.59E-09 | 1.26E-07 | UP |
| SNORA71C | 1.142243962 | 1.33E-08 | 2.83E-07 | UP |
| SNORD14E | 1.224152307 | 2.85E-08 | 5.63E-07 | UP |
| SNORD60 | 1.030902058 | 3.10E-08 | 5.97E-07 | UP |
| SNORD36C | 0.999407876 | 4.28E-08 | 7.67E-07 | UP |
| SNORA38B | 0.628722055 | 1.37E-07 | 2.41E-06 | UP |
| SNORA60 | 0.871151074 | 1.98E-06 | 2.94172E-05 | UP |
| SNORD72 | 0.679270627 | 1.52E-06 | 2.29912E-05 | UP |
| SCARNA12 | 0.842319656 | 7.39E-06 | 9.40027E-05 | UP |
| snoZ196 | 0.783158313 | 8.12E-06 | 0.000101719 | UP |
| SNORA71B | 0.778686016 | 1.12959E-05 | 0.000139257 | UP |
| SNORA14A | 0.704227595 | 2.12E-06 | 3.10047E-05 | UP |
| ENSG10010138531.1 | 0.861624012 | 3.63E-06 | 5.11326E-05 | UP |
| SNORD1B | 0.591213218 | 6.27E-06 | 8.25161E-05 | UP |
| SNORD12B | 0.788817785 | 3.85525E-05 | 0.000422471 | UP |
| ENSG10010139899.1 | 0.7006634 | 0.000153689 | 0.001595534 | UP |
| SNORA5A | 0.652476072 | 0.000177935 | 0.001799878 | UP |
| SNORA11 | 0.617493841 | 0.000829544 | 0.006817813 | UP |
| SNORD19 | 0.596888413 | 0.001988146 | 0.015229588 | UP |
| SNORA12 | 0.652479454 | 0.020079779 | 0.043765044 | UP |
| ENSG10010137877.1 | -1.323498365 | 1.50E-39 | 2.96E-37 | DOWN |
| SNORA80D | -2.126214942 | 6.46E-31 | 7.28E-29 | DOWN |
| ENSG10010134187.1 | -3.886783481 | 4.77E-31 | 6.28E-29 | DOWN |
| ENSG10010136902.1 | -1.778335997 | 3.85E-20 | 3.04E-18 | DOWN |
| SNORA9B | -1.13589534 | 3.95E-14 | 2.08E-12 | DOWN |
| SNORD116-4 | -2.324558897 | 1.22E-13 | 5.35E-12 | DOWN |
| SNORD59A | -0.868565302 | 5.22E-13 | 2.17E-11 | DOWN |
| SNORA22B | -0.641677381 | 3.39E-11 | 1.12E-09 | DOWN |
| SNORA63C | -1.239710901 | 8.13E-11 | 2.47E-09 | DOWN |
| ENSG10010136194.1 | -1.468247363 | 8.91E-10 | 2.20E-08 | DOWN |
| SNORA24B | -0.851317835 | 2.22E-08 | 4.49E-07 | DOWN |
| ENSG10010138828.1 | -1.143727568 | 4.14E-08 | 7.59E-07 | DOWN |
| ENSG10010140115.1 | -0.659641287 | 1.55E-07 | 2.66E-06 | DOWN |
| SNORA77 | -1.078991324 | 7.69E-07 | 1.21286E-05 | DOWN |
| ENSG10010136505.1 | -0.81083636 | 4.80E-06 | 6.63945E-05 | DOWN |
| SNORD89 | -0.669708605 | 5.12E-06 | 6.96554E-05 | DOWN |
| ENSG10010136290.1 | -0.757720756 | 5.78E-06 | 7.73433E-05 | DOWN |
| SNORA70B | -1.247817413 | 3.66196E-05 | 0.000406941 | DOWN |
| ENSG10010137222.1 | -0.631100495 | 0.000225819 | 0.002140995 | DOWN |
| ENSG10010135879.1 | -1.625956809 | 0.006653318 | 0.027480157 | DOWN |
| SNORA2C | -0.654810769 | 0.006832766 | 0.027480157 | DOWN |
| SNORA79B | -0.704177677 | 0.008466574 | 0.027480157 | DOWN |
| ENSG10010134689.1 | -1.650247612 | 0.010780091 | 0.027480157 | DOWN |
| ENSG10010135148.1 | -0.702301948 | 0.003631768 | 0.024479008 | DOWN |
