# Supplementary material for: Identification of Small Nucleolar RNA SNORD60 as a Potential Biomarker and Its Clinical Significance in Lung Adenocarcinoma
Source: Biomed Res Int. 2022 Jun 7;2022:5501171. doi: 10.1155/2022/5501171 (PMC9197630; doi:10.1155/2022/5501171)
Supplement: Supplementary Materials — Supplementary Table S1: the demographic and clinicopathological characteristics of TCGA LUAD cohort. Supplementary Table S2: the clinicopathologic characteristics of LUAD patients. Supplementary Table S3: amplification sequences in quantitative RT-PCR. Supplementary Table S4: differential expression snoRNAs of LUAD TCGA database were detected by R packages “edgeR”. Supplementary Table S5: differential expression snoRNAs of LUAD TCGA database were detected by R packages “limma.” Supplementary Table S6: snoRNA profiling in three matched surgically resected LUAD tissues. Supplementary Figure S1: a screenshot of the file filter settings in TCGA website (https://portal.gdc.cancer.gov). Supplementary Figure S2: the relationship between SNORD60 expression and other clinical factors (including age, sex, pathological T category, and distant metastasis). Supplementary Figure S3: Kaplan-Meier analysis of overall survival. There was no difference between the high and low SNORD60 expression groups (P > 0.05). The median expression level of SNORD60 was used as the cutoff. [file 5501171.f1.zip › tableS6.doc]

Table S6. snoRNA Profiling in three matched surgically resected LUAD tissues

| **Name** | **Fold Difference** | ***P*-value** | **change** |
| --- | --- | --- | --- |
| SCARNA1 | 4.150 | 1.513E-2 | UP |
| SNORA78 | 3.395 | 2.562E-2 | UP |
| SNORA10 | 2.135 | 2.142E-2 | UP |
| SNORA40 | 2.054 | 1.320E-2 | UP |
| SNORD60 | 1.524 | 4.147E-2 | UP |
| SNORA60 | 0.214 | 7.414E-3 | DOWN |
| SNORD115-28 | 0.322 | 4.788E-2 | DOWN |
| SNORA12 | 0.440 | 4.408E-2 | DOWN |
| SNORA20 | 0.464 | 1.647E-2 | DOWN |
| SNORD77 | 0.618 | 2.453E-2 | DOWN |
